# Real-World 12-Month Outcomes with Sirolimus-Coated Balloon Angioplasty for Complex Femoropopliteal Disease

**DOI:** 10.3390/jcm14020483

**Published:** 2025-01-14

**Authors:** Efthymios Beropoulis, Konstantinos Avranas, Elena Rouvi, Konstantinos P. Donas

**Affiliations:** 1 Department of Vascular and Endovascular Surgery, Rhein Main Vascular Center, Asklepios Clinics Langen, Paulinen Wiesbaden, 63225 Langen, Germany; eberopoulis@gmail.com; 2 Research Collaborator Rhein Main Vascular Center, Department of Cardiology, Asklepios Clinic Langen, 63225 Langen, Germany; avranaskon@gmail.com; 3 Research Collaborator Rhein Main Vascular Center, 63225 Langen, Germany; elenarouvi29300@yahoo.gr

**Keywords:** peripheral arterial disease, sirolimus-coated balloon, endovascular intervention, vessel preparation, critical limb threatening ischemia, real-world evidence

## Abstract

**Background**: Sirolimus-coated balloons (SCBs) have emerged as a promising alternative to paclitaxel-coated devices for the treatment of femoropopliteal lesions. However, real-world data on SCB performance in also complex peripheral arterial disease remains unknown. We sought to evaluate the safety and 12-month clinical outcomes of the Selution SLR™ balloon angioplasty in a challenging real-world patient cohort. **Methods**: This single-center, retrospective observational study with prospective follow-up included 21 patients with symptomatic peripheral arterial disease treated with the Selution SLR™ SCB (Med. Alliance, SA, Mont-sur-Rolle, Switzerland) after vessel preparation with rotational atherectomy, between October 2023 and November 2024. The primary endpoints were technical success, 12-month primary patency, and target lesion revascularization (TLR). Secondary endpoints included major adverse cardiac events (MACE), major adverse limb events (MALE), and changes in Rutherford classification and ankle-brachial index (ABI). **Results**: The median age was 79 years, with 47.6% of patients over 80 years old. Most patients presented with advanced peripheral atherosclerotic disease (PAD) (Rutherford category V, 47.6%). Lesions were predominantly occlusive (76.2%), with a median length of 130 mm and severe/moderate calcified in 71.4% of cases. Technical success was achieved in 95.2% of procedures. The 12-month primary patency was 95%, with a TLR-Rate of 5%. No major amputations or cardiovascular deaths occurred. Significant improvements in Rutherford category and ABI were maintained at 12 months. **Conclusions**: In this real-world cohort of patients with complex PAD, vessel preparation-assisted Selution SLR™ angioplasty demonstrated safety and promising 12-month outcomes. These findings support the use of SCBs in also challenging peripheral interventions, though larger-scale data and further follow up are needed in order to establish SCBs’ role as crucial in the treatment algorithm of PAD.

## 1. Introduction

Peripheral arterial disease (PAD) represents a significant healthcare burden, with atherosclerotic lesions in the femoropopliteal axis being the predominant cause of lifestyle-limiting claudication and critical limb threatening ischemia (CLTI). Over the past decade, endovascular interventions have emerged as the primary treatment strategy for these patients, with drug-coated devices playing a pivotal role in improving outcomes.

Paclitaxel (PTX)-coated balloons demonstrated superior anti-restenotic properties compared to plain old balloon angioplasty (POBA), significantly reducing clinically driven target-lesion revascularization (TLR) rates in femoropopliteal segments [1,2]. However, in December 2018, a meta-analysis of randomized controlled trials suggested an increased mortality risk in PAD patients treated with paclitaxel-coated devices [3]. Despite subsequent analyses of patient-level data and longer-term outcomes showing diminished strength of this adverse mortality signal [4,5], these safety concerns prompted the development of alternative drug-coated devices.

Sirolimus emerged as a promising alternative, building upon extensive experience with sirolimus-coated stents and balloons in coronary interventions. From a pharmacological perspective, sirolimus demonstrates cytostatic and anti-inflammatory effects with a broader therapeutic window [6]. However, the successful transfer of sirolimus into the arterial wall presents technical challenges that required innovative solutions [7].

Currently, two sirolimus-coated balloons have received approval to be investigated within randomized studies for symptomatic PAD treatment: The Magic-Touch™ (Concept Medical, Tampa, FL, USA) and the Selution SLR™ (Med. Alliance, SA, Mont-sur-Rolle, Switzerland). Early clinical evidence from single-arm trials demonstrated promising 6-month results, with freedom from angiographic restenosis exceeding 90% in femoropopliteal lesions [8]. Several randomized trials are ongoing but data especially in complex PAD are scant.

Real-world data on the performance of sirolimus-coated balloons in routine clinical practice remains unknown. Therefore, this study aims to evaluate the safety and feasibility of the Selution balloon in real-life practice among consecutive patients with symptomatic PAD undergoing endovascular treatment.

## 2. Methods

### 2.1. Study Design and Patient Population

This study focused on patients with symptomatic peripheral arterial disease (PAD) requiring endovascular intervention, as first line treatment.

We therefore included patients presenting with Rutherford category II–V symptomatology, with lesions located in the native superficial femoral artery (SFA) or proximal popliteal artery. Patients presenting with Rutherford class VI symptoms, such as acral necrosis or gangrene, were excluded, as clinical outcomes in these cases are significantly influenced by local wound management. Eligible lesions had a stenosis of ≥70% or complete occlusion, as assessed by visual angiography. The vessel diameter ranged from 4 to 6 mm, and the total lesion length was between 30 and 210 mm. All patients were treated exclusively with one or more sirolimus-coated balloons. The use of debulking devices was not an exclusion criterion.

This study included only patients with no prior treatment of the target lesion, excluding those who had previously undergone debulking procedures, balloon angioplasty, or stent placement. Limbs with previous bypass treatment were excluded. Additionally, patients on dialysis were excluded from the study.

Early procedural and 12-month clinical data of patients treated with the Selution SLR™ sirolimus-coated balloon (Med. Alliance, SA, Mont-sur-Rolle, Switzerland) between October 2023 and November 2024 were prospectively collected and retrospectively evaluated. The study was conducted in accordance with the Declaration of Helsinki, and the evaluation was approved by our local ethics committee (2023-3323-evBO). All patients provided written informed consent for the procedure and analysis.

### 2.2. Pre-Procedural Assessment

All patients underwent a comprehensive clinical examination, including assessment of peripheral pulses, skin temperature, capillary refill, and overt signs of ischemia such as pallor, ulcers. As mentioned, Rutherford VI patients were excluded. Additionally, cardiovascular risk factors and comorbidities such as hypertension, dyslipidemia or smoking history were documented for each patient followed by the measurement of the Ankle-Brachial Index (ABI). Arterial duplex ultrasound was performed to identify lesions. The Rutherford classification system was used to categorize the severity of peripheral arterial disease (PAD).

### 2.3. Endovascular Procedure

All procedures were performed under loco-regional anesthesia with conscious sedation if needed. Access site selection (antegrade or retrograde) was based on lesion characteristics and operator preference. After obtaining arterial access, intravenous unfractionated heparin (70–100 IU/kg) was administered.

Lesion crossing was attempted using standard wire and catheter techniques. After successful crossing intraluminally, vessel preparation by use of rotational atherectomy to debulk the lesion was used in all included cases, followed by sirolimus-coated balloon. The selection of it was based on quantitative vascular analysis with 10% oversizing relative to the reference vessel diameter. The deployment protocol included:

- Balloon preparation according to manufacturer specifications
- Inflation time of 180 s at nominal pressure per lesion segment for each individual balloon
- Single inflation per lesion segment
- Overlap margins of at least 5 mm when multiple balloons were required

Bailout stenting was permitted in cases of flow-limiting dissection or residual stenosis and recoil > 50% Post-procedural medication included dual antiplatelet therapy (aspirin 100 mg/day and clopidogrel 75 mg/day) for 3 months followed by single antiplatelet therapy indefinitely. In order to evaluate the reproducibility of the performance, we categorized the colleagues who treated the included cases into either “young” or “experienced” endovascular surgeons. In case of certificate of “endovascular specialist” from the German Society of Vascular Medicine and Vascular Surgery, we defined the colleagues as experienced endovascular surgeons.

### 2.4. Outcome Measures and Definitions

#### 2.4.1. Primary Endpoints

Technical success was defined as achievement of <30% residual stenosis without flow-limiting dissection. Primary patency was defined freedom from restenosis (>50%) or occlusion and Target Lesion Revascularization (TLR) rate at 12 months.

#### 2.4.2. Secondary Endpoints

Major adverse cardiac events (MACE) included a composite of cardiovascular death, non-fatal myocardial infarction and ischemic stroke. As Major adverse limb events (MALE) was defined a composite of major amputation and target vessel revascularization.

### 2.5. Follow-Up Protocol

Patients were evaluated at 1, 3, 6, and 12 months through clinical assessment, including palpation of peripheral pulses and measurement of the ankle-brachial index (ABI). Additional visits were scheduled if clinically or anamnestic indicated. Loss to follow-up was minimized through a dedicated study nurse coordinator and telephone contact system.

### 2.6. Statistical Analysis

Data analysis was performed using SPSS Version 28 (IBM Corp., Armonk, NY, USA). Continuous variables were expressed as median with interquartile range (IQR). Categorical variables were presented as frequencies and percentages. Survival analyses for primary patency and freedom from TLR were performed using Kaplan-Meier estimates. Due to the non-normal distribution of the data and the limited sample size, non-parametrical tests were deployed. A *p*-value < 0.05 was considered statistically significant.

## 3. Results

### 3.1. Patient and Lesion Characteristics

Between October 2023 and November 2024, 21 from overall 283 patients with symptomatic PAD underwent endovascular treatment with the Selution SLR™ sirolimus-coated balloon. As shown in Table 1, the study population included 8 males (38.1%) with a median age of 79 years (IQR: 74.5–84.5, with nearly half (47.6%) being over 80 years old. The clinical presentation was predominantly advanced disease, with most patients presenting in Rutherford category V (47.6%), followed by category IV (28.6%) and category III (23.8%). The median baseline ankle-brachial index was 0.5 (IQR: 0.4–1.3).

The cardiovascular risk profile of our cohort, detailed in Table 2, demonstrated a high prevalence of comorbidities. Hypertension was the most common risk factor, present in 76.2% of patients. Pre-existing coronary artery disease and chronic kidney disease were present in 23.8% and 9.5% of patients, respectively. Pre-procedure antithrombotic medication included aspirin in 52.4% of patients, while fewer patients were on clopidogrel (4.8%) or direct oral anticoagulants (14.3%).

### 3.2. Lesion and Procedural Characteristics

**Figure 1:**
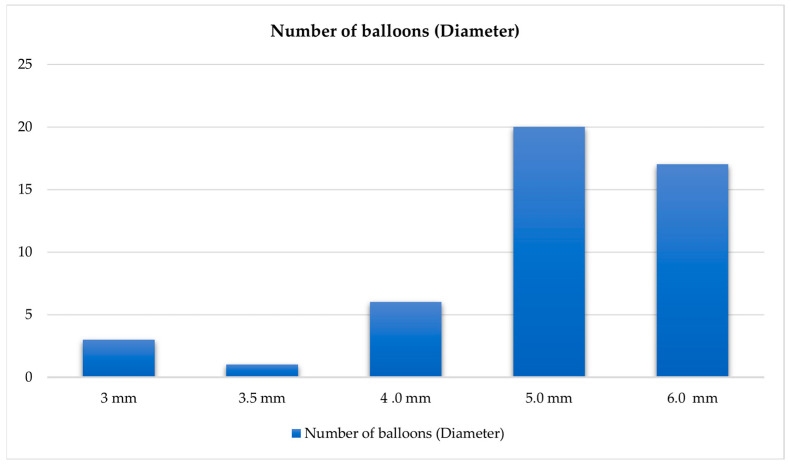
Diameter of balloons.

**Figure 2:**
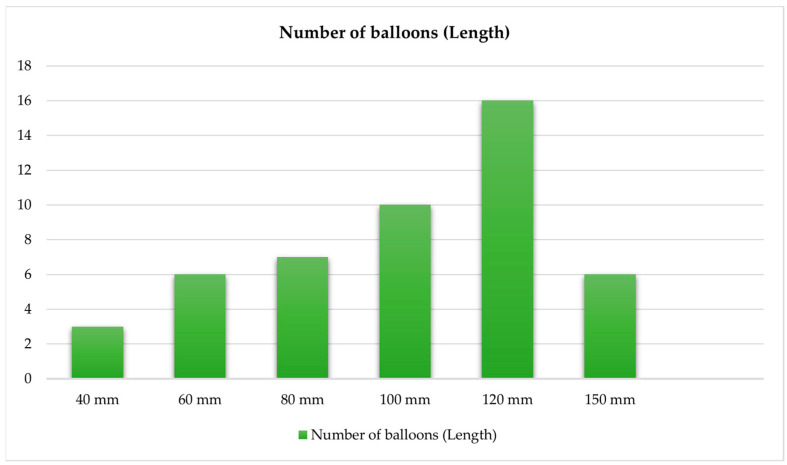
Length of balloons.

The interventional characteristics of our cohort are presented in Table 3. A total of 26 vessel segments were treated, with occlusive disease predominating over stenotic lesions (76.2% vs. 23.8%). The treated lesions showed considerable complexity, with a median length of 130 mm (IQR: 67.5–225.0 mm) and varying degrees of calcification. Severe and moderate calcification (25–100% of vessel circumference) was present in 71.4% compared to mild (<25%) calcification in 28.6% of the treated cases, respectively. The distal runoff pattern showed two-vessel runoff in 42.9% of cases, followed by three-vessel (33.3%) and single-vessel runoff (23.8%). Right-sided limb procedures accounted for 38.1% of interventions. Figure 1 and Figure 2 illustrate diameter and size used of the balloons.

### 3.3. Procedural Details and Technical Outcomes

The retrograde cross-over approach was predominantly utilized (85.7%) compared to antegrade access (14.3%), with a median procedure duration of 104 min (IQR: 87.0–120.0). The interventions required a total of 48 sirolimus-coated balloons, with a median of 2 balloons per treated segment (IQR: 1–2). Distal protection devices were selectively employed in 14.3% of procedures.

Technical success was achieved in 95.2% of cases. Bailout stenting was necessary in 8 procedures, predominantly due to flow-limiting dissections, with one case requiring stenting for residual stenosis exceeding 50%. The maximum stent length deployed was 150 mm. Periprocedural complications were observed in two cases: one distal embolization occurred after the debulking successfully managed with aspiration thrombectomy and local thrombolysis, and one side branch below-the-knee perforation completely resolved with prolonged balloon inflation.

### 3.4. Early Outcomes and Post-Procedural Care

The immediate post-procedural period was marked by one minor amputation. As documented in Table 4, the post-procedural antithrombotic regimen was intensified, with aspirin prescribed to 85.7% of patients, clopidogrel to 61.9%, and direct oral anticoagulants to 38.0%. The median ABI showed improvement to 0.9 (IQR: 0.85–1.30), *p* < 0.01.

Young operators required longer procedure times (median 118 min vs. 92 min for experienced operators, *p* = 0.04) and showed a higher tendency to use bailout stenting (35.7% vs. 22.2%, *p* = 0.08).

### 3.5. Twelve-Month Follow-Up Outcomes

**Figure 3:**
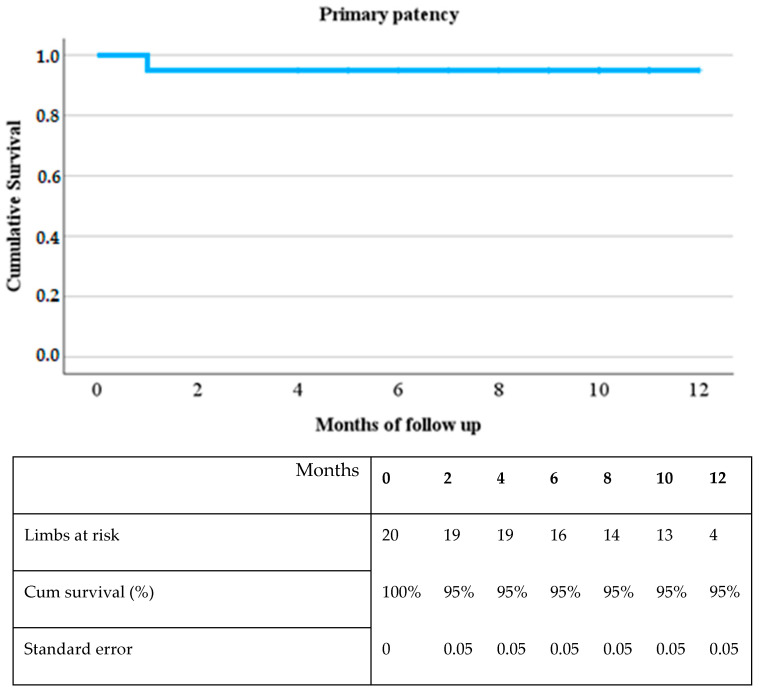
Kaplan Meier Curve of the primary patency with a targeted follow-up of 12 months.

One-year follow-up data were available for 20 patients, excluding one case without technical success. The Kaplan-Meier analysis, presented in Figure 3, demonstrates excellent 12-month patency. One early occlusion occurred within the first month, attributed to an initially undetected dissection, and was successfully managed with thrombus aspiration and stenting. Following this event, the cumulative survival rate remained stable at 95% through the 12-month follow-up period, with secondary patency maintained at 100%.

Clinical outcomes were favorable, with only one MACE event recorded—a transient ischemic attack following gastrointestinal bleeding. No cardiovascular deaths or major amputations occurred during the follow-up period. TLR was required in only one case. Clinical improvement was evident through sustained enhancement in both Rutherford categories and ABI, with 85% of patients showing improvement by two or more Rutherford categories.

## 4. Discussion

To our knowledge, this data represents the first time in the literature of a 12-month real-world experience with atherectomy-assisted Selution SCB which demonstrated excellent technical success rate of 95.2% despite complex lesion characteristics, promising 12-month primary patency and low TLR rate of 5.0%. Use of atherectomy-assisted SCB was safe with minor periprocedural complications. These results are particularly noteworthy given our challenging patient population, characterized by advanced age (median 79.0 years), high prevalence of cardiovascular risk factors, and complex lesion morphology with predominantly occlusive disease (76.2%) and significant calcification (71.4% moderate to severe).

Several factors may contribute to this promising early experience of atherectomy-assisted SCB angioplasty. First, the vessel preparation in all cases in term of a standardized deployment protocol, including mandatory 180-s inflation times, provides clear procedural guidance. Moreover, the device’s handling characteristics appear similar to conventional balloons, minimizing the additional technical complexity.

Recent trial data provide important context to our findings. The recently published SELUTION SFA Japan trial [9] enrolled 134 patients and demonstrated 12-month primary patency of 87.9% and freedom from TLR of 97.0%. While their results align with ours, the treated cohort in Japan had clearly shorter and less calcified and complex lesions compared to our included cases.

The PRISTINE registry [10] provides valuable insights into SCB performance in 75 patients with critical limb-threatining ischemia (CTLI). The primary patency of 74.0% at 6 months and 58.0% at 12 months, with freedom from clinically-driven TLR of 84.0% and 74.0% respectively, were slightly inferior than in our study. However, their population included a high proportion of patients with diabetes (91%) and end-stage renal failure (37%).

The PRESTIGE trial [11,12] specifically evaluated Selution SLR™ in BTK interventions for CLTI patients. Their 12-month data showed sustained efficacy with maintained target lesion patency and freedom from TLR (92.6%), along with excellent wound healing rates (85.7%). This durability of effect is particularly noteworthy given the challenging and quite controversial nature of drug-coated BTK interventions in the CLTI setting.

A key advantage of sirolimus-coated technology appears to be the absence of the “slow-flow phenomenon” that has been observed with paclitaxel-coated devices. As demonstrated in recent comparative studies by Tang et al. [13], sirolimus-eluting balloons showed no evidence of slow-flow, whereas both high-dose and low-dose paclitaxel platforms exhibited varying degrees of reduced flow after application. This difference may be particularly important in CLTI patients with limited vascular reserve and poor outflow vessels.

The pharmacological profile of sirolimus offers several potential theoretical advantages over paclitaxel. Recent review data [14] highlights that sirolimus has a 100-fold higher margin of safety and broader therapeutic range compared to paclitaxel. Furthermore, sirolimus’s cytostatic rather than cytotoxic action, combined with its anti-inflammatory properties, may contribute to a more favorable vascular healing response. The successful 12-month clinical outcomes in our series suggest that the Selution SLR™’s proprietary microreservoir drug delivery technology adequately addresses the historical challenge of efficient sirolimus transfer to the arterial wall, even in complex lesion subsets.

The evolution from paclitaxel to sirolimus-based devices must be considered in the context of safety concerns raised by Katsanos et al. [3] regarding long-term mortality with paclitaxel devices. Our study’s safety findings, albeit in a limited sample size, support the favorable safety profile of sirolimus-coated technology, with no mortality events and minimal complications during follow-up.

The role of vessel preparation, particularly through atherectomy [15,16], emerged as a critical factor in procedural success. Young operators who followed a systematic approach to lesion preparation, including careful assessment of calcification and appropriate use of atherectomy devices, achieved comparable technical success rates to their more experienced colleagues. The atherectomy-first strategy provided several advantages such as improved luminal gain, enhanced drug delivery through better vessel compliance, and reduced risk of flow-limiting dissections. This finding supports the implementation of structured training programs emphasizing the importance of adequate lesion preparation, especially for complex, calcified lesions.

Several limitations of our study should be acknowledged. Our investigation was conducted at a single center with a relatively small sample size, which inherently limits the broad generalizability of our findings to other clinical settings, different types of sirolimus-eluting balloons and patient populations. While our 12-month follow-up period provided valuable insights into mid-term efficacy, this timeframe may be insufficient to capture late restenotic events or potential longer-term safety signals that could emerge beyond the first year of treatment. The single-arm design of our study, lacking a control group, prevents direct comparative analysis with alternative treatment strategies, making it challenging to definitively position this therapy within the current treatment paradigm. Furthermore, although we maintained rigorous clinical follow-up, our protocol did not include routine angiographic surveillance, which may have led to underestimation of the true restenosis rate, particularly in asymptomatic patients. Finally, selection bias, attrition bias and confounding, as well as risk of type II statistical error should be noted.

Looking ahead, several critical questions remain to be addressed. The ongoing SIRONA trial [17] comparing sirolimus versus paclitaxel-coated balloons will provide essential comparative efficacy data. The role of sirolimus-coated balloons in below-the-knee interventions is being further investigated in multiple trials, including SELUTION4BTK (NCT05055297) and PRESTIGE-BTK. Additionally, future studies should focus on optimizing lesion preparation strategies for heavily calcified vessels, potentially incorporating intravascular imaging guidance.

## 5. Conclusions

Our findings suggest that atherectomy-assisted sirolimus-coated balloon angioplasty represents a promising therapeutic option for complex femoropopliteal disease in real-world practice. The favorable safety profile and encouraging efficacy data support its role as an alternative to paclitaxel-based platforms. The absence of slow-flow phenomenon and sustained drug delivery characteristics may be particularly advantageous in complex lesions. However, larger-scale, long-term data from real-world registries and randomized trials comparing different drug-coated technologies will be essential to definitively establish its place in the endovascular treatment algorithm for PAD.

## Figures and Tables

**Table 1:** Demographical and baseline data.

**Demographical data**	
Age (years)	79 (74.5, 84.5)
Age over 80 years old	10 (47.6%)
Sex (male)	8 (38.1%)
**Baseline data**	
Rutherford Category	
II	4 (3.5, 5.0)
III	5 (23.8%)
IV	6 (28.6%)
V	10 (47.6%)
ABI	0.5 (0.4, 1.3)

**Table 2:** Comorbidities and medication.

**Comorbidities**	
Smoking history	11 (52.4%)
Hypertension	16 (76.2%)
Dyslipidemia	14 (66.7%)
Diabetes	12 (57.1%)
Coronary Artery Disease	5 (23.8%)
Chronic Kidney Disease	2 (9.5%)
**Medication**	
Aspirin	11 (52.4%)
Clopidogrel	1 (4.8%)
Direct Oral Anticoagulant	3 (14.3%)

**Table 3:** Interventional data.

Interventional Data	
Young operator	10 (47.6%)
Operation duration (min)	104 (IQR: 87.0–120.0).
Type of lesion	
Stenosis	5 (23.8%)
Occlusion	16 (76.2%)
Lesion Length (mm)	100 (IQR: 67.5, 225.0)
Number of run-off vessels	
I	5 (23.8%)
II	9 (42.9%)
II	7 (33.3%)
Grade of calcification	
0–25%	6 (28.6%)
25–50%	5 (23.8%)
50–75%	7 (33.3%)
75–100%	3 (14.3%)
Limb side (right leg)	8 (38.1%)
Type of access	
Antegrade	3 (14.3%)
Retrograde	18 (85.7%)

**Table 4:** Postinterventional data.

Postinterventional Data	
ABI	9 (IQR: 8.5, 13.0)
Aspirin	18 (85.7%)
Clopidogrel	13 (61.9%)
Direct Oral Anticoagulant	8 (38%)

## Data Availability

The original contributions presented in this study are included in the article. Further inquiries can be directed to the corresponding author.
